# Cancer beliefs and screening behaviors: The impact of neighborhood and other social determinants of health

**DOI:** 10.3389/fonc.2023.1072259

**Published:** 2023-01-27

**Authors:** Tracy M. Layne, Parul Agarwal, Bruce D. Rapkin, Lina H. Jandorf, Nina A. Bickell

**Affiliations:** ^1^ Departments of Population Health Science and Policy, and Obstetrics, Gynecology, and Reproductive Science, the Blavatnik Family Women’s Health Research Institute and the Center for Scientific Diversity at the Icahn School of Medicine at Mount Sinai, New York, NY, United States; ^2^ Department of Population Health Science and Policy, Icahn School of Medicine at Mount Sinai, New York, NY, United States; ^3^ The Tisch Cancer Institute, Icahn School of Medicine at Mount Sinai, New York, NY, United States; ^4^ Department of Epidemiology and Population Health, Albert Einstein College of Medicine, New York, NY, United States; ^5^ Department of Family and Social Medicine, Albert Einstein College of Medicine, New York, NY, United States

**Keywords:** cancer screening, breast cancer, colorectal cancer, community outreach, social determinansts of health

## Abstract

**Background:**

Beliefs about cancer influence breast and colorectal cancer (CRC) screening behavior. Screening rates for these cancers differ in the contiguous neighborhoods of East Harlem (EH), Central Harlem (CH), and the Upper East Side (UES), which have distinct socio-demographic compositions. We assessed the belief-screening behavior relationship in these neighborhoods.

**Methods:**

The 2019 Community Cancer Needs Survey included adults eligible for breast and/or colorectal cancer screening. Raking was used to generate neighborhood-specific distribution estimates. Categorical variables were compared using Chi-square tests. Stepwise logistic regression models were used to estimate odds ratios (OR) and 95% confidence intervals (CI) for the association between cancer beliefs and screening.

**Results:**

Our weighted sample included 147,726 respondents. Screening was 75% in CH, 81% in EH, and 90% in the UES for breast cancer, and 71%, 76%, and 92% for CRC, respectively. The fatalistic belief *“There’s not much you can do to lower your chances of getting cancer”* differed by neighborhood with screening more likely in CH respondents (breast OR =1.45 and colorectal OR =1.11), but less likely in EH (OR= 0.77 and 0.37, respectively). UES ORs were not generated due to too few unscreened respondents.

**Conclusions:**

Cancer beliefs were inconsistently associated with breast and CRC screening across three NYC neighborhoods. This suggests that a given belief may either motivate or deter screening, depending upon context or interpretation. Once access is addressed, efforts seeking to enhance screening rates should consider implications of communities’ varying beliefs.

## Introduction

Where we live can affect our lives’ trajectories (1). Its impact on environmental exposures, social and cultural realities, and the access and availability of services is well described (1–5). Less is known, however, about the impact of neighborhood on cancer beliefs and cancer screening behaviors, though prior research suggests variability across geographic regions distinguished by socioeconomic status (SES) and geographic isolation (6). The influence of neighborhood on cancer beliefs, screening, and health behaviors is relevant as cancer centers seek to better characterize and address the cancer prevention and control needs of their catchment areas (7), and as these areas expand to better capture geographic locales where patients live.

In the current study, we consider neighborhood in the context of cancer screening for breast and colorectal (CRC) cancers for which there are concrete recommendations (8, 9) and evidence that beliefs influence behavior (10–12). Screening rates for these cancers differ within and across the richly diverse neighborhoods of New York City (NYC). Here, we focus on Central Harlem (CH), East Harlem (EH), and the Upper East Side (UES) – contiguous NYC neighborhoods bordering our cancer center that vary in their racial and ethnic, SES, other social determinants of health compositions, and their cancer incidence and mortality rates (13). The latter is evident in the higher odds of developing cancer overall associated with living in CH or EH compared to the UES (14). For CRC, the age-standardized rate of new cases is higher in CH (43.3 per 100,000) and EH (41.4) compared to the UES (28.8) but lower for breast cancer in CH (144.5) and EH (129.7) than the UES (164.4) (15). The age-standardized mortality rate, however, is higher in the Harlem neighborhoods compared to the UES for both CRC (CH 39.7 per 100,000 and EH 35.8 vs. 23.6), and breast cancer (75.3 and 56.7 vs.42.3 (16).

Given the known differences in the distribution of racial/ethnic groups, SES, and breast and colorectal cancer outcomes across these neighborhoods, we examined the relationship between six Health Information Trends Survey (HINTS) cancer beliefs which capture respondents’ cancer risk perceptions (17, 18), including beliefs about cancer fatalism and screening, both overall and by neighborhood. We also assessed the relationship between sociodemographic factors, medical mistrust, and healthcare access with cancer screening behavior.

## Materials and methods

A random sample of participants were recruited to complete the Icahn School of Medicine at Mount Sinai Community Cancer Needs Survey from two sources: 1) the Mount Sinai Health System electronic medical record (EMR) (N=598), including 18% with a history of cancer based on International Classification of Diseases coding; and 2) community outreach to the Tisch Cancer Institute at the Icahn School of Medicine catchment areas of CH, EH, and the UES (N = 604). Participants were eligible if they were ≥ age 18, spoke either Spanish or English, were able to provide informed consent, and resided in the following neighborhoods based on zip code: CH (zip codes: 10026, 10027, 10030, 10037, 10039), EH (zip codes: 10029, 10035), and the UES (zip codes: 10128, 10021, 10044, 10065, 10075). For recruitment, our target neighborhood distribution was 40% (N=500) each from EH and CH, and 20% from UES (N=200) to ensure strong representation from vulnerable communities. The original unweighted sample included 1,202 participants total, with 480 (40%) from CH, 498 (41%) from EH, and 224 (19%) from the UES.

Participants identified from the EMR were recruited using hard copy and email invitations during the first two months of recruitment. Thereafter, email invite was used given the similar response rate of ~3% across methods. Community outreach participants were recruited from faith-based organizations, health centers, community development and social service organizations, street fairs, parks, storefronts (e.g., supermarkets), public housing, subway and bus stops. All respondents took a 45-minute survey, either assisted or online; and received a $20 gift card for participation. Surveying occurred from April to September 2019.

### Survey measures and cancer beliefs

The survey measured domains of: socio-demographics (e.g., age, gender, race/ethnicity, income, education, insurance), cancer screening, cancer beliefs, health information seeking behavior and access, healthcare access, health history, family history of cancer, general health status, and medical mistrust. In addition to HINTS, we used validated items from national surveys (i.e., Behavioral Risk Factor Surveillance System, National Health Interview Survey) (19, 20) as well as newly created or modified questions resulting in a 167-item survey.

We examined six cancer beliefs (17, 18), including four fatalistic questions: 1) *“It seems like everything causes cancer”*, 2) *“There is not much you can do to lower your chances of getting cancer”* 3) *“There are so many different recommendations about preventing cancer, it’s hard to know which ones to follow”*, 4) *“When I think of cancer I automatically think of death”*; and two non-fatalistic belief questions: 1) “*Cancer is most often caused by a person’s behavior or lifestyle*”, and 2) “*I’d rather not know my chances of getting cancer.*” All beliefs had the following responses: 1=strongly agree, 2=somewhat agree, 3=somewhat disagree and 4=strongly disagree. In analyses, we compared those who “agree” (combination of responses 1 and 2) to those who “disagree” (combination of 3 and 4). Mistrust was measured using a 6-item Group-based Medical Mistrust scale (21), with response values ranging 1= strongly agree to 5 strongly disagree, and scored (range 6-30) such that lower scores indicated greater mistrust.

### Breast and colorectal cancer screening outcomes

Recommended screening was defined as having a mammogram within the past 2 years for women ≥40 years for breast cancer, and having blood stool screening in the past year or colonoscopy in the past 10 years for men and women ≥50 years for CRC.

### Statistical analyses

We aimed to recruit individuals representing the census distribution for each neighborhood, however, our final sample distribution was not adequately representative. To obtain better representation of the base population, we combined the EMR and community data sources and then raked the entire dataset, applying population-based weights using data from NYC Health Atlas (22) to obtain estimates based on a cross-classification of age-sex-race-ethnicity-neighborhood factors. Raking, also known as sample-balancing, is an iterative post stratification method that weights the individual survey responses such that the marginal proportions of the survey approximate those of the base population (23, 24). Specifically in this iterative and sequential process, each row of the cross-classified factors are weighted so that the sample row totals are consistent with the totals of the base population. Next, each column of these data are similarly adjusted so that the column totals align with column totals of the base population (24). As a post stratification method, raking is thought to reduce nonresponse bias of the sample data, thereby improving the quality of the sample data (25). However, we acknowledge that raking does not account for or provide an unbiased sample for certain health factors (e.g., access to care) that may differ based on recruitment of participants from the EMR versus the community. We compared categorical variables using Chi-square tests, and estimated odds ratios (OR) and 95% confidence intervals (CI) for stepwise logistic models of the association between beliefs and receipt of screening and used P <0.25 as the threshold for retention in the model. For the forward stepwise analyses, we entered the following factors into the models: age, race/ethnicity, marital status, income, insurance, medical mistrust, general health status, usual source of routine care, difficulty understanding health care provider due to participant’s language, personal and family history of cancer, and cancer beliefs. Tables below include final model-specific factors obtained from stepwise regression.

The breast cancer screening model resulted in inconclusive results when all beliefs were entered into the model simultaneously. As such, the following two beliefs were excluded, as they were not statistically significant when evaluated with all other beliefs: “*I’d rather not know my chances of getting cancer* “and “*When I think of cancer I automatically think of death”*.

Multivariable models examining the cancer belief-cancer screening relationship by neighborhood were not feasible for all beliefs or for all three neighborhoods due to the lack of convergence for the UES. This is largely due to the relatively low number of UES respondents who did not receive recommended screening. As such, multivariable models of the cancer belief-cancer screening association were only examined for CH and EH. For the EH CRC screening model, we did not enter usual source of routine care as a covariate because 95% of the analytic sample had access to care. We also replaced income with education in the same model as only one individual in the unweighted data had an annual household income ≥$75,000 (the referent category) who did not adhere to recommended screening guidelines. As such, we could not generate sufficient weighted data for comparisons made in this particular analysis.

All analyses were conducted using Statistical Analysis Software (SAS) version 9.4.

This study was approved by the institutional review board of the Icahn School of Medicine at Mount Sinai.

## Results

Descriptive factors, for the overall weighted sample (N=147,726) and each neighborhood, are summarized in **Table 1**. Looking at the latter breakdown, respondents in CH and EH were younger (56 and 57 years, respectively), compared to those in the UES (64 years), had lower annual household income (52% and 55% <$35K, respectively, compared to 10% in the UES) and education (39.7% and 38.6% with high school education or less, respectively, compared to about 2% in the UES), and a larger proportion were uninsured (10% in CH and 7% in EH vs. about 2% in the UES). In terms of the racial/ethnic majorities in each neighborhood, respondents were largely non-Hispanic Black (59%) in CH, Hispanic in EH (46%), and non-Hispanic White in the UES (90%).

**Table 1:** Descriptive characteristics of survey respondents to a Community Cancer Needs Survey in Central Harlem, East Harlem, and the Upper East Side.

	Overall N=147,726		Central Harlem N=58,901		East Harlem N=54,055		Upper East Side N=34,770				
Weighted N and %	N		%	N	%	N	%		N		%
Age, Mean (min, max)	58 (40, 92)		56 (40, 91)		57 (40, 91)		64 (41, 92)				
Gender											
Female	76,609		51.9	31,617	54	26,369	49		18,623		54
Male	71,117		48.1	27,284	46	27,686	51		16,147		46
Race/Ethnicity											
Hispanic	38,039		25.7	11,552	20	24,889	46		1,598		5
Non-Hispanic White	49,717		33.7	9,666	16	8,722	16		31,329		90
Non-Hispanic Black	50,126		33.9	34,757	59	15,144	28		225		1
Other	9,844		6.7	2,926	5	5,301	10		1,618		5
Neighborhood											
Central Harlem	58,901		39.9	Not applicable							
East Harlem	54,055		36.6								
Upper East Side	34,770		23.5								
Annual Household Income											
$0-$34,999	63,792		43.2	30,624	52	29,629	55		3,540		10
$35,000 - $74,999	25,719		17.4	11,107	19	10,532	19		4,080		12
$75,000 or more	48,011		32.5	13,358	23	10,566	20		24,086		69
Missing	10,204		6.9	3,812	6	3,328	6		3,064		9
Education											
High School (HS) or less	44,810		30.3	23,407	40	20,846	39		557		2
vocational training or some college	28,288		19.1	12,296	21	12,698	23		3,294		9
college graduate	32,571		22	11,275	19	10,911	20		10,385		30
postgraduate	41,265		27.9	11,335	19	9,601	18		20,329		58
Missing	793		0.5	588	1	0	0		205		1
Insurance											
Employer or Union	49,050		33.2	19,344	33	15,721	29		13,985		40
Medicaid or Other State Program/Exchange	40,161		27.2	18,227	31	17,602	33		4,332		12
Medicare	43,381		29.4	12,548	21	15,539	29		15,294		44
Other	2,959		2	2,000	3	754	1		205		1
No insurance	10,413		7	6,123	10	3,767	7		524		2
Missing	1,762		1.2	658	1	673	1		431		1
General Health Status											
Excellent/Very good	69,617		47.1	27,015	46	23,289	43		19,313		56
Good	41,881		28.4	17,666	30	16,305	30		7,909		23
Fair/Poor/Very poor	35,406		24	13,805	23	14,053	26		7,547		22
Missing	823		0.6	415	1	408	1		0		0
A place usually go for routine or preventive care											
Yes	130,943		88.6	50,046	85	47,786	88		33,110		95
No - there is no place I usually go for routine or preventive care	13,608		9.2	8,143	14	4,841	9		625		2
Missing	3,176		2.1	712	1	1,428	3		1,035		3
How often feel like you do not understand your health provider because of your language											
Always/Often//frequently/sometimes	33,935		23	17,770	30	14,610	27		1,555		4
Never	108,364		73.4	37,788	64	38,117	71		32,460		93
Missing\Don’t know\ Don’t remember	5,427		3.7	3,343	6	1,329	2		755		2
Medical mistrust (1 = higher mistrust 5 = lower mistrust), Mean (min, max)	4 (1,5)		3.8(1,5)		3.9 (1,5)		4.6 (1,5)				
	Overall N=147,726		Central Harlem N=58,901		East Harlem N=54,055		Upper East Side N=34, 770				
Weighted N and %	N	%	N	%	N		%	N		%	
Personal history of cancer											
Yes	27,455	18.6	6,730	11	10,032		19	10,693		31	
No	119,423	80.8	51,967	88	43,379		80	24,077		69	
Missing	848	0.6	204	0	644		1	0		0	
Family (any) history of cancer											
Yes	99,024	67	35,314	60	35,602		66	28,108		81	
No/Not sure	47,830	32.4	23,435	40	17,733		33	6,662		19	
Missing	873	0.6	152	0	721		1	0		0	
CANCER BELIEFS											
*It seems like everything causes cancer*											
Strongly agree/Somewhat agree	76,838	52	32,696	56	32,469		60	11,673		34	
Somewhat disagree/Strongly disagree	67,099	45.4	25,050	43	19,642		36	22,407		64	
Missing	3,790	2.6	1,155	2	1,945		4	690		2	
There’s not much you can do to lower your chances of getting cancer											
Strongly agree/Somewhat agree	40,283	27.3	20,153	34	14,897		28	5,234		15	
Somewhat disagree/Strongly disagree	103,505	70.1	37,041	63	37,477		69	28,986		83	
Missing	3,939	2.7	1,707	3	1,682		3	550		2	
*There are so many different recommendations about preventing cancer, it’s hard to know which ones to follow*											
Strongly agree/Somewhat agree	96,402	65.3	38,443	65	35,614		66	22,345		64	
Somewhat disagree/Strongly disagree	48,558	32.9	19,152	33	17,326		32	12,080		35	
Missing	2,767	1.9	1,306	2	1,116		2	345		1	
When I think of cancer I automatically think of death											
Strongly agree/Somewhat agree	77,425	52.4	32,630	55	28,148		52	16,647		48	
Somewhat disagree/Strongly disagree	67,417	45.6	24,999	42	24,985		46	17,433		50	
Missing	2,885	2	1,272	2	922		2	690		2	
Cancer is most often caused by a person’s behavior or lifestyle											
Strongly agree/Somewhat agree	52,641	35.6	20,034	34	22,584		42	10,023		29	
Somewhat disagree/Strongly disagree	90,506	61.3	36,356	62	29,952		55	24,197		70	
Missing	4,580	3.1	2,510	4	1,520		3	550		2	
I’d rather not know my chances of getting cancer											
Strongly agree/Somewhat agree	46,566	31.5	21,098	36	15,245		28	10,223		29	
Somewhat disagree/Strongly disagree	97,540	66	36,418	62	36,920		68	24,202		70	
Missing	3,621	2.5	1,385	2	1,891		3	345		1	
CANCER SCREENING											
Breast cancer screening among women ≥40 years											
Yes, mammography ≤ 2 years ago	61,980	80.9	23,752	75	21,422		81	16,807		90	
Yes, mammography >2 years ago/Never	12,816	16.7	6,892	22	4,108		16	1,816		10	
Missing	1,813	2.4	973	3	840		3	0		0	
Colorectal cancer screening among men and women ≥50 years											
Yes, blood stool screen in past year or colonoscopy in past 10 years	80,857	78.9	27,649	71	26,570		76	26,638		92	
Yes ever/Never	20,917	20.4	11,061	28	7,771		22	2,085		7	
Missing	726	0.7	130	0	391		1	205		1	

Additionally, a lower proportion of respondents in CH and EH reported their general health status as “excellent” or “very good health” (46% and 43% respectively), relative to those in the UES (56%). While most in all three neighborhoods reported a source of routine care (≥85% for all neighborhoods), difficulty understanding a health provider due to the respondents language was greater in CH (30%) and EH (27%) compared to the UES respondents (4%), and medical mistrust scores indicated greater mistrust in the Harlem neighborhoods (3.8 in CH and 3.9 in EH) relative to the UES (4.6). A lower proportion of respondents in CH and EH reported both a personal and family history of cancer compared to respondents in the UES. In terms of cancer beliefs, respondents in CH and EH reported more agreement with fatalistic cancer beliefs relative to those in the UES. With regard to screening, 75% of CH women reported having breast cancer screening, compared to 81% in EH and 90% in the UES, compared to 74% previously reported for NYC overall (26). For CRC screening, the distribution was 71% in CH, 77% in EH and 92% in the UES, compared to 69% previously reported among NYC the 69% noted here applies to all adults 50 and over, not just women overall (27).

**Table 2** summarizes the multivariable modeling results for the relationship between four cancer beliefs and recommended breast cancer screening. Women who agreed “*It seems like everything causes cancer*” were more likely to be screened compared to those that disagreed (OR = 1.09, 95% CI: 1.04-1.15). A similar positive association was observed for those we agreed there are “*too many recommendations, hard to know what to follow*” (OR = 1.12, 95% CI: 1.07-1.18); and “*Cancer is most often caused by behavior or lifestyle*” (OR = 1.35, 95% CI: 1.29-1.42). The latter belief had the strongest point estimate of the belief-screening behavior associations examined. Women who agreed “*There’s not much you can do to lower your chances of getting cancer*” were less likely to be screened compared to those who disagreed with this fatalistic cancer belief. Women with less medical mistrust had a greater likelihood of screening (OR for every incremental increase in the score = 1.23, 95% CI: 1.20-1.26). Compared to non-Hispanic White women, Hispanic and non-Hispanic Black women were less likely to be screened in adjusted models, while women of Other race/ethnicity, which includes those from Asian/Pacific Islander, American Indian/Alaska Native, and multiracial backgrounds, were more likely than their White counterparts to be screened.

**Table 2:** Multivariable logistic regression for association between cancer beliefs, and other factors, with recommended breast cancer screening among women ≥40 years.

	Odds Ratio^†^	95% Confidence Interval		P-value
Cancer Beliefs (Agree vs. Disagree)				
*It seems like everything causes cancer*	1.09	1.04	1.15	0.0004
*There’s not much you can do to lower your chances of getting cancer*	0.73	0.70	0.77	<.0001
*There are so many different recommendations about preventing cancer, it’s hard to know which ones to follow*	1.12	1.07	1.18	<.0001
*Cancer is most often caused by a person’s behavior or lifestyle*	1.35	1.29	1.42	<.0001
Age	1.01	1.01	1.01	<.0001
Race-Ethnicity (Reference = Non-Hispanic White)				
Hispanic	0.74	0.69	0.79	<.0001
Non-Hispanic Black	0.51	0.48	0.55	<.0001
Other Race/Ethnicity	1.29	1.16	1.44	<.0001
Married vs Other	1.10	1.05	1.16	0.0002
Annual Household Income (Reference = ≥$75,000)				
$0-$34,999	0.95	0.88	1.03	0.2012
$35,000 - $74,999	1.06	0.99	1.14	0.0971
Insurance (Reference = Medicaid)				
Employer or Union	1.23	1.15	1.31	<.0001
Medicare	1.58	1.47	1.69	<.0001
Other Insurance	1.70	1.43	2.02	<.0001
No Insurance	0.54	0.50	0.60	<.0001
Medical Mistrust	1.23	1.20	1.26	<.0001
General Health Status (Reference = Fair/Poor)				
Excellent/Very Good	1.60	1.52	1.69	<.0001
Good	1.25	1.18	1.32	<.0001
A place usually go for routine or preventive care (Reference = Yes)				
No	0.33	0.30	0.36	<.0001
How often feel like you do not understand your health provider because of your language (reference = Never)^‡^				
Ever	^§^			
History of Cancer (Reference = No)				
Personal history of cancer	1.10	1.04	1.16	0.001
Family (any) history of cancer	^§^			

^†^Odds ratio for the outcome of recommended breast cancer screening: Yes, mammography ≤ 2 years ago vs. Yes, mammography >2 years ago/Never. Model covariates include all items listed in the table except where indicated.

^‡^Ever = Always/Often/Frequently/Sometimes.

^§^The stepwise regression model eliminated this variable.

**Table 3** summarizes the multivariable modeling results for the relationship between all six cancer beliefs and recommended CRC screening. Among women and men age ≥50 years eligible for screening, most fatalistic cancer beliefs were associated with a reduced likelihood of screening. Here again, the strongest association was the belief “*Cancer is most often caused by behavior or lifestyle*”, though in the opposite direction than observed for breast cancer (OR = 0.59, 95% CI: 0.56-0.61). Less medical mistrust was similarly associated with a higher likelihood of CRC screening, while Hispanic, NH-Black, and Other race/ethnicity was associated with a reduced likelihood compared to those that are NH-White.

**Table 3:** Multivariable logistic regression for association between cancer beliefs, and other factors, with recommended colorectal cancer screening among women and men ≥50 years.

	Odds Ratio^†^	95% Confidence Interval		P-value
Cancer Beliefs (Agree vs. Disagree)				
*It seems like everything causes cancer*	0.72	0.68	0.75	<.0001
*There’s not much you can do to lower your chances of getting cancer*	0.79	0.76	0.83	<.0001
*There are so many different recommendations about preventing cancer, it’s hard to know which ones to follow*	0.80	0.76	0.84	<.0001
*Cancer is most often caused by a person’s behavior or lifestyle*	0.59	0.56	0.61	<.0001
*I’d rather not know my chances of getting cancer*	0.85	0.81	0.89	<.0001
*When I think of cancer I automatically think of death*	1.31	1.25	1.37	<.0001
Age	^‡^			
Gender	^‡^			
Race-Ethnicity (Reference = Non-Hispanic White)				
Hispanic	0.47	0.43	0.50	<.0001
Non-Hispanic Black	0.68	0.63	0.72	<.0001
Other Race/Ethnicity	0.28	0.26	0.31	<.0001
Married vs Other	1.79	1.70	1.88	<.0001
Annual Household Income (Reference = ≥$75,000)				
$0-$34,999	0.60	0.56	0.65	<.0001
$35,000 - $74,999	0.49	0.45	0.53	<.0001
Insurance (Reference = Medicaid)				
Employer or Union	2.57	2.40	2.76	<.0001
Medicare	1.86	1.77	1.95	<.0001
Other Insurance	0.84	0.75	0.93	0.0014
No Insurance	0.44	0.41	0.48	<.0001
Medical Mistrust	1.05	1.02	1.07	<.0001
General Health Status (Reference = Fair/Poor)				
Excellent/Very Good	1.70	1.62	1.79	<.0001
Good	1.42	1.35	1.50	<.0001
A place usually go for routine or preventive care (Reference = Yes)				
No	0.67	0.63	0.72	<.0001
How often feel like you do not understand your health provider because of your language (Reference = Never)^§^				
Ever	0.85	0.81	0.89	<.0001
History of cancer (Reference = No)				
Personal history of cancer	1.40	1.32	1.48	<.0001
Family (any) history of cancer	1.29	1.24	1.35	<.0001

^†^Odds ratio for the outcome of recommended breast cancer screening: Yes, mammography ≤ 2 years ago vs.Yes, mammography >2 years ago/Never. Model covariates include all items listed in the table except where indicated.

^‡^The stepwise regression model eliminated this variable.

^§^Ever = Always/Often/Frequently/Sometimes.

### Neighborhood

Results for the evaluation of beliefs in CH and EH are shown in **Table 4**.The belief that cancer is most often due to a person’s behavior or lifestyle was associated with a lower odds of recommended cancer screening for breast and CRC in both neighborhoods, though the association was strongest for CRC among EH respondents (OR = 0.42). A similar pattern and magnitude of association was evident for the belief “*It seems like everything causes cancer”* for both cancer screening outcomes and across neighborhood (OR range = 0.71- 0.74), though no estimate could be generated for CRC screening in EH. For both screening outcomes, the belief “*There’s not much you can do to lower your chances of getting cancer*” was consistently associated with a higher odds of screening in CH, but a lower odds of screening in EH. For all other beliefs, where estimates could be generated, the associations varied by neighborhood and cancer screening type. Notably, results by neighborhood from multivariable models included adjustment for race/ethnicity.

**Table 4:** Multivariable logistic regression for association between cancer beliefs with recommended colorectal cancer screening in CH and EH.

Cancer Beliefs	Breast Cancer Screening		Colorectal Cancer Screening	
	Odds Ratio^†^			
	CH^‡^	EH^§^	CH^¶^	EH^††^
*It seems like everything causes cancer*	0.72	0.71	0.74	N/A
*There’s not much you can do to lower your chances of getting cancer*	1.45	0.77	1.11	0.37
*There are so many different recommendations about preventing cancer, it’s hard to know which ones to follow*	1.22	1.62	0.47	2.25
*When I think of cancer I automatically think of death*	^‡‡^		1.82	1.90
*Cancer is most often caused by a person’s behavior or lifestyle*	0.68	6.58	0.81	0.42
*I’d rather not know my chances of getting cancer*	^‡‡^		0.53	0.91

^†^Odds ratio for the outcome of recommended breast cancer screening: Yes, mammography ≤ 2 years ago vs. Yes, mammography >2 years ago/Never. Model covariates include the following: age; race/ethnic (Hispanic, Non-Hispanic Black, and Non-Hispanic White (referent); annual household income ($0-$34,999, $35,000-$74,999, and ≥$75K (referent); insurance (private (employer/union), public (Medicare and Medicaid), and Other (referent); medical mistrust; general health status (Excellent/Very Good, Good, and Fair/Poor (referent)); usual source of routine care (no vs. yes); difficulty understanding health care provider (always/often, frequently/sometimes vs. never); every had cancer (yes vs. no) family history of cancer (any) (yes vs. no); marital status (married, others (referent)).

^‡^Stepwise regression eliminated marital status and difficulty understanding health care provider from this model.

^§^Stepwise regression eliminated difficulty understanding provider because of language from this model Same as Model a with no eliminations in the stepwise regression.

^††^Same as Model a except stepwise regression eliminated marital status. Usual source of routine care was not added to this model as 95% of the analytic sample had access to care.

^‡‡^This belief was excluded due to inconclusive results obtained when simultaneously entered into the model with all beliefs.

## Discussion

This analysis found that cancer beliefs inform guideline concordant screening behaviors for breast and CRC, and that there are important underlying socio-demographic and neighborhood-level differences in the relationships that require further study. Interestingly, we observed the strongest overall belief-screening behavior association for those that believe cancer is mostly due to behavior or lifestyle; which was associated with an increased likelihood of screening for breast cancer, but decreased likelihood for CRC. These findings highlight important opportunities for cancer centers to create cancer-specific screening interventions that are responsive to the nuanced needs and influences in a given catchment area.

HINTS cancer belief questions similar to those used in the current study have also linked cancer beliefs to cancer screening behavior for breast (10, 11) and CRC screening (28). For mammography among caregivers – defined as those providing care or making decisions for someone with a disability, or health or behavioral condition – those who would rather not know the likelihood of getting cancer were less likely to be screened compared to those that disagreed (11). In a separate study among Asian Americans, cancer fatalism was found to be a predictor of screening adherence for breast and cervical cancers (10), but non-adherence for CRC (12, 28). A prior analysis using four of the HINTS cancer belief questions used in the current study found that CRC fatalism was higher in Asians and Hispanic respondents vs. Whites (28). However, after adjustment for sociodemographic, health status and access information, and fatalistic CRC beliefs, Asians were more likely to adhere to CRC screening compared to White respondents (OR = 2.04) (28). The opposite pattern of association was found among Hispanic respondents, however, such that they were less likely to adhere to CRC compared to White respondents after adjustment for socio-demographic factors and fatalistic cancer beliefs (OR = 0.90) (28). Taken together, findings from prior studies – and our own – suggests SES and culture (29, 30) may have variable influence on cancer beliefs both across and within (10, 12) racial/ethnic groups, and that these beliefs differently influence cancer screening behavior (10). Cancer screening campaigns targeting neighborhoods where these groups reside will need to consider such nuances, as a one-size fits all approach will not address the cancer prevention and control needs of these communities.

The relationship between health beliefs and cancer screening behavior has been examined among racial and ethnic minority groups (12, 28, 29), finding racial and ethnic differences in cancer beliefs (10, 28, 31–33), cancer screening behavior (12, 28, 29), and variations in the association between beliefs and cancer screening behavior across these groups (10, 11, 28, 34–36). The independent and combined influence of socio-demographic factors, such as race and SES, have also been found to be important predictors of cancer beliefs (31). Prior studies have evaluated factors associated with health seeking behavior and health care utilization (37). However, such studies (36, 37) have not consistently captured other relevant factors that impact screening and health seeking behavior such as, access to health care (e.g., insurance), language barriers, demographic, and SES factors (37). This is meaningful given substantial research documenting differences in beliefs across race and SES, with the former having a stronger influence. In a study assessing four of the HINTS questions used in the current study, Black race was directly associated with negative cancer beliefs independent of and beyond SES as measured by income and educational attainment. Notably, SES only partially mediated the relationship between Black race and negative cancer beliefs (31). In the current study, however, associations between cancer beliefs with breast and CRC screening were independent of both race/ethnicity and SES factors.

Geographical differences in cancer beliefs and perceptions have also been observed. Appalachian states differed significantly from a nationally represented sample based on HINTS data on four of five HINTS cancer beliefs examined in the current study (6). Overall, these findings point to variations in cancer beliefs across, and within segments of the population that will be important to understand to meaningfully encourage and sustain cancer control and prevention efforts. This is particularly true in geographic areas defined by considerable differences in race, ethnicity, and SES, as is the case for the three distinct neighborhoods examined in the current study.

Targeted initiatives can successfully engage and improve outcomes. This was true of the NCI’s Colorectal Cancer Outreach and Screening Initiative, which increased both awareness, connection to care, and CRC screening in a national sample of racially, ethnically, and culturally diverse groups (38). In addition, identifying factors relevant across the cultural and socio-politically heterogeneous communities that makeup racial/ethnic subgroups (e.g., Hispanic/Latinx communities) will likely have the greatest impact on improving the cancer prevention and control disparities observed among them (39). These findings highlight the importance of truly targeted outreach. Successful engagement with different communities requires cancer centers to develop sensitive and specific approaches to outreach that take into account the influence of culture, beliefs, and sociodemographic factors on behaviors, including cancer screening. Our analysis of New York City neighborhoods with distinct racial, ethnic, and socioeconomic profiles demonstrates the need for more granularity in community needs assessments to help inform cancer prevention and control.

In the current study, we sought to better understand what drove the differential cancer belief-screening behaviors associations across Central and East Harlem. Specifically, we reexamined the dataset –weighted and unweighted data – to identify potential neighborhood-specific differences that might explain the observed findings. We found no evidence of errors, nor did reexamination help explain the observed differences. Our findings may instead reflect a lack of linearity in beliefs, such that a given belief can be both a motivator and barrier to screening in a particular context, or in this case, neighborhood. Additional research, particularly qualitative studies, are needed to directly assess and unpack the predictors of the likely intersection of cancer beliefs and screening behavior.

Limitations of this study include a low response rate among those recruited *via* U.S. mail and e-mail for those recruited through the electronic medical record, and the inability to assess response rates at the community level. Our ability to model neighborhood effects was limited due to the high correlations of sociodemographic factors and neighborhood. However, this feature of our dataset highlights the importance of capturing key differences of populations within a cancer center’s purview. In NYC, a city famous for its multi-cultural populace and close proximity of diverse peoples, identifying such differences and addressing them may hold the key to advancing equitable cancer care. Further, this was a cross-sectional study and we are unable to ascribe cause and effect of beliefs with screening behaviors (40, 41). Cancer screening rates across the two Harlem neighborhoods evaluated were relatively similar and this lack variability in screening rates may have limited our ability to detect meaningful differences in the cancer beliefs-screening behavior relationship across neighborhoods, particularly for those with lower screening rates. Rates of screening behaviors were based on self-report, which have been described as an accurate measure (40, 42, 43). Strengths include use of validated survey items to access cancer beliefs as well as factors relevant to the community’s awareness and needs as it relates to cancer services; these survey instruments also allowed for comparison with prior findings. Additionally, we used statistical methods (i.e., raking and weighting) to expand the representativeness of our data to align with the distributions found in the examined neighborhoods. Finally, this study adds to the understanding of the role of cancer beliefs in screening behavior by considering previously studied socio-demographic factors along with neighborhood dynamics. Our findings are consistent with prior research identifying differences in cancer perceptions and beliefs in rural vs. non-rural communities (6); all of which suggests that cancer belief assessments may be valuable tools for better understanding barriers and facilitators of cancer screening in these communities.

Our findings suggest that targeted initiatives to increase cancer screening need to consider structural impediments (e.g., access to care), as well as community-specific beliefs about cancer that influence behavior. Such initiatives might include using data obtained from the regular assessment of community-level cancer beliefs to inform the development of cancer screening awareness materials and advertisements, as well as campaigns designed to connect the community to cancer screening opportunities. In the next phase of this work, larger studies are needed to expand the evaluation across neighborhoods to understand how this environment and its characteristics – the settings in which communities cultivate their beliefs, behaviors, and health – influence cancer beliefs. Investments towards understanding communities, particularly those at high risk for poor cancer outcomes, through such work will better inform development of equitable approaches to improving screening and other cancer detection and control objectives.

## Data availability statement

The raw data supporting the conclusions of this article will be made available by the authors, without undue reservation.

## Ethics statement

The studies involving human participants were reviewed and approved by The Institutional Review Board of the Icahn School of Medicine at Mount Sinai. The participants provided their written informed consent to participate in this study.

## Author contributions

TL: Conceptualization, formal analysis, data curation, and writing – original draft and review and editing. PA: Formal analysis, methodology, data curation, and review and editing. BR: Methodology, review and editing. LJ and NB: Conceptualization, supervision, funding acquisition, and review and editing. All authors contributed to the article and approved the submitted version.
